# Elicitation-Based Method for Increasing the Production of Antioxidant and Bactericidal Phenolic Compounds in *Dionaea muscipula* J. Ellis Tissue

**DOI:** 10.3390/molecules25081794

**Published:** 2020-04-14

**Authors:** Wojciech Makowski, Krzysztof Michał Tokarz, Barbara Tokarz, Rafał Banasiuk, Karolina Witek, Aleksandra Królicka

**Affiliations:** 1 Department of Botany, Physiology and Plant Protection, Faculty of Biotechnology and Horticulture, University of Agriculture in Krakow, 29 Listopada 54, 31-425, Krakow, Poland; km.tokarz.ipbb@gmail.com (K.M.T.); barbara.tokarz@urk.edu.pl (B.T.); karolina11880@vp.pl (K.W.); 2 Institute of Biotechnology and Molecular Medicine, Trzy Lipy 3, 80-172 Gdansk, Poland; banasiuk@herbiopharm.pl; 3 Intercollegiate Faculty of Biotechnology UG and MUG, Laboratory of Biologically Active Compounds, University of Gdansk, Abrahama 58, 80-307 Gdansk, Poland

**Keywords:** antibiotic-resistant bacteria, biotic elicitation, *Cronobacter sakazakii*, DPPH, *Escherichia coli*, plant secondary metabolites, *Staphylococcus aureus*, Venus Flytrap

## Abstract

The carnivorous plant *Dionaea muscipula* J. Ellis (Venus flytrap) is a widely known medical herb, capable of producing various phenolic compounds known for their strong antioxidant and antibacterial properties. In the pharmaceutical industry, Venus flytrap is grown in tissue cultures, as the natural population of *D. muscipula* is very limited. Here, we describe an improved method to increase the quantity and quality of phenolic compounds produced in *D. muscipula.* This is achieved by combining biotic elicitation (using *Cronobacter sakazakii* bacteria lysate) of *D. muscipula* cultured with rotary shaking (hydromechanical stress), which we describe here for the first time. The antibacterial activity and the antioxidant properties of the obtained compounds were studied on two antibiotic-resistant human pathogenic bacteria. The proposed plant culture conditions resulted in an increase in fresh weight, as well as a higher total phenolic content, in comparison to traditional tissue cultures on agar-solidified medium. With the use of high-performance liquid chromatography, we demonstrated that the described elicitation strategy leads to an increased synthesis of myricetin, caffeic acid, ellagic acid and plumbagin in *D. muscipula* tissue. We also found that a higher level of antioxidant activity, exhibited by the plant extract, corresponded with its higher phenylpropanoid content. The bactericidal activity of the extract against *Staphylococcus aureus* was dependent on the duration of plant culture under described elicitation conditions, whereas neither elicitation condition (duration or elicitor concentration) seemed relevant for the bactericidal activity of the extract towards *Escherichia coli*. This suggest that Gram-negative bacteria are less sensitive to compounds derived from Venus flytrap tissue.

## 1. Introduction

Carnivorous plants from the family, Droseraceae, have been used in natural medicine around the world for centuries. The first known report on the application of *Drosera* sp. herb in natural therapy is dated in 12th century [1]. The medical properties of these plants arise from the ability to synthesize various secondary metabolites from a group of phenolic compounds, especially 1,4-naphtoquinones derivatives, among which plumbagin (PLU) is the most common derivative [2,3]. Due to the chemical structure of PLU (5-hydroxy-2-methyl-1,4-naphthoquinone), this compound can undergo redox cycling and generate reactive oxygen species (ROS) in cells, resulting in its biological activity [2].

According to Gaascht et al. [2], secondary metabolites are highly diversified and complex group of plant derived chemicals, accumulated in very small amount [4], with various biological activities. As shown, the extracts from carnivorous plants from the family, Droseraceae, have strong antioxidant [5], antibacterial [1,6] and antifungal [7] properties. Recently, Kawiak et al. [8] showed that they also have anticancer properties. Due to the growing resistance for antibiotics of some human-pathogenic microbes, and the increasing demand for new drugs in cancer therapy, plant material with strong biological activity are in great demand. 

The main source of PLU in the medical plants industry are in field growing *Plumbago* sp. plants, although characterized by the low concentration of secondary metabolites [9]. On the other hand, *Plumbago* sp. is known to be a heavy metals accumulator [10], so the root material from the natural environment can be contaminated and toxic. Furthermore, *D. muscipula* plants grown in vitro can accumulate between 15–20 times more PLU per gram of biomass than the *Plumbago* roots culture [11,12,13,14]. Despite this, *D. muscipula* is not using as an industrial source of PLU, because of limited availability of plant material.

In the family, Droseraceae, the most abundant in phenolic compounds and rich in PLU is *Dionaea muscipula* J. Ellis (Venus flytrap) [2,3]. This plant grows in a marshy, wet, nutrient-poor and sun-exposed areas, in North and South Carolina of the United States, being an endemit and the only species in genus *Dionaea* [13]. To survive in a natural environment, *D. muscipula* synthesizes phenolic compounds to protect against predators, UV radiation and putrefaction processes during digestion of prey [15]. Phenolic compounds are produced via shikimate/phenylpropanoid or polyketide acetate-malonate pathways and play a crucial role in plant-environment interactions [16]. The natural population of *D. muscipula* is very small, and obtaining plants from natural habitats is impossible. To create an environment-independent source of this valuable plant material, in vitro propagation protocols have been established [17]. Tissue culture technique makes it possible to obtain large amounts of plant biomass in a short time. The most common technique for in vitro plant propagation is the cultivation of whole plants or plant organs on solid medium with agar. However, modifications of this basic technique, such as culture in liquid media (LM) [18] or temporary immersion bioreactors [19,20], allow for greater plant biomass or plant-derived compounds production. In this study, for the first time, we show how cultivation of the Venus flytrap in LM with rotary shaking affects the plant’s secondary metabolites accumulation and biomass production, in comparison to traditional solid medium (SM)—experiment 1. We hypothesised that, because of the physical features of LM and shaking, the plant will have better accessibility to medium resources and better conditions for efficient growth. On the other hand, rotary shaking can be a mechanical stress factor for *D. muscipula* and leads to increased synthesis of some phenolic compounds. Based on the results and observations from experiment 1, we have designed conditions for an experiment with elicitation—experiment 2.

Elicitation is a plant biotechnology technique based on exogenous addition of elicitors (biotic or abiotic) to the growth medium. This is one of the most effective ways to increase plant secondary metabolites production [21] and biological activity of plant-derived extract [1], although some medical plants are not always sensitive to elicitation [14,20]. Elicitors are perceived by specific receptors in the plasma membrane, trigger gene expression and induce a stress response in plants, which may result in production of higher amounts or new forms of valuable secondary metabolites [9]. However, stress modifies plant metabolism, growth or reproduction in a negative and/or positive way [16]. Stress response can be manifested on various levels of plant organization and usually has unspecific character, so elicitation strategies do not always work effectively and should be investigated.

To the extent of our knowledge, there are a few studies focused on elicitation strategies of *Drosera* sp. plants using various elicitors [18,22,23,24], but little is known about the possible elicitation strategies for *D. muscipula* [1]. Due to the unique biochemical compositions and strong biological activity of Venus flytrap extract, studies on this species are valuable. Our previous study on this plant showed that abiotic elicitation with a blue-red LED light did not increase synthesis of PLU and did not affect total phenolic content (TPC) [14]. In this experiment, we examined the response of Venus flytrap in vitro plants grown in LM with rotary shaking to elicitation with lysate of *Cronobacter sakazakii* (former *Enterobacter sakazakii*) bacteria. This is the first report showing such an elicitation strategy, where for the first time, an elicitor has been used in a concentration and time gradient. Moreover, we present how such elicitation affects the antioxidant properties and antibacterial activity against two antibiotic-resistant human-pathogenic bacteria: *Staphyloccocus aureus* and *Escherichia coli.*

Bacterial-derived elicitors are proven to stimulate plant secondary metabolism in plant tissue cultures, and the preparation of these is easy and fast compared to other biotic elicitors, e.g., fungal derived elicitors [21]. *C. sakazakii* lysate has been chosen for our experiment based on literature data for other plant species [20,25]. *C. sakazakii* are the human-pathogenic, facultative, Gram-negative, non-spore forming and motile microorganisms (possess flagella) that belong to the *Gammaproteobacteria* class and the Enterobacteriaceae family [26]. Flagellin was found to be the main, if not the only, factor in the recognition of Gram-negative bacteria (e.g., various pathovars *Pseudomonas syringae*, *E. coli*, *Pseudomonas aeruginosa*) by plant cells [27]. Moreover, specific bacterial O-polysaccharides [28] can be a signal for the plant cell, which will trigger a stress response and will affect higher secondary metabolites production. Furthermore, elicitation in LM with rotary shaking may turn out to be a good system for elicitation studies, due to easier and faster elicitor action. 

The aims of the study were; (1) evaluation of biometric and biochemical parameters of *D. muscipula* plants growing in LM with rotary shaking, (2) optimisation of elicitation protocol using lysate from *C. sakazakii* bacteria cells in the context of synthesis of medical active phenolic compounds belonging to 1,4-naphtoquinones, phenolic acids, phenylpropanoids, flavonoids and anthocyanins, (3) check antioxidant and antibacterial properties of extracts isolated from elicited plant tissue.

Present research indicate that LM system is more suitable for *D. muscipula* plant cultivation, than agar-solidified media. Elicitation with *C. sakazakii* lysate causes changes in biochemical composition of examined plants, and increase its biological activity against reactive oxygen species and antibiotic-resistance bacteria.

## 2. Results

### 2.1. Biometric and Biochemical Parameters of Plants Growing in LM (Experiment 1)

**Figure 1:**
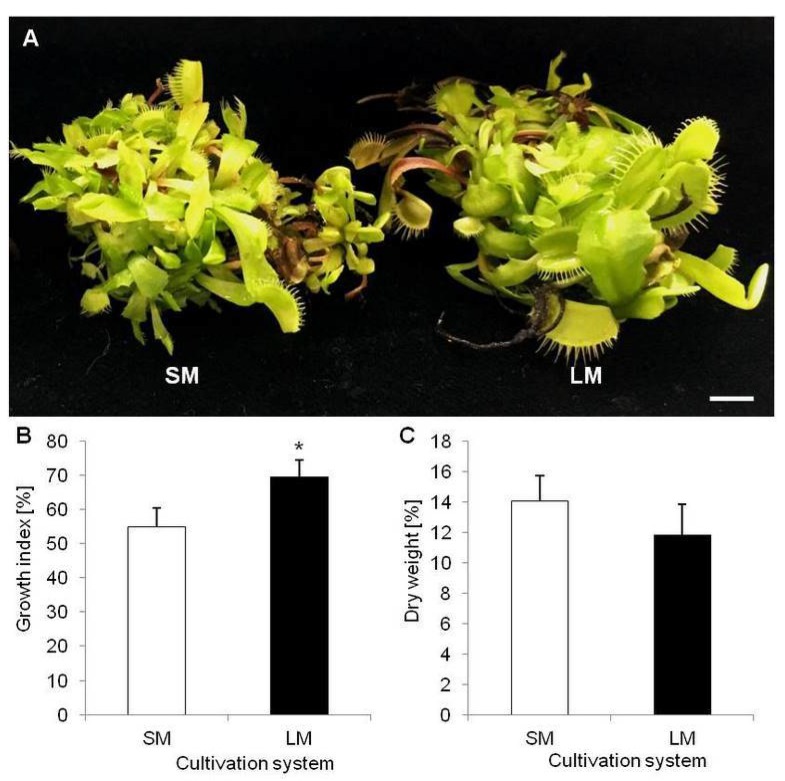
*Dionaea muscipula* plants in different cultivation systems: (**A**) plants cultivated on solid medium (SM) and in liquid medium with rotary shaking (LM); (**B**) growth index [%] of plants depending on cultivation system; (**C**) dry weight content [%] of plants depending on cultivation system; * significant difference between means at *p* < 0.05; bar—1 cm.

**Figure 2:**
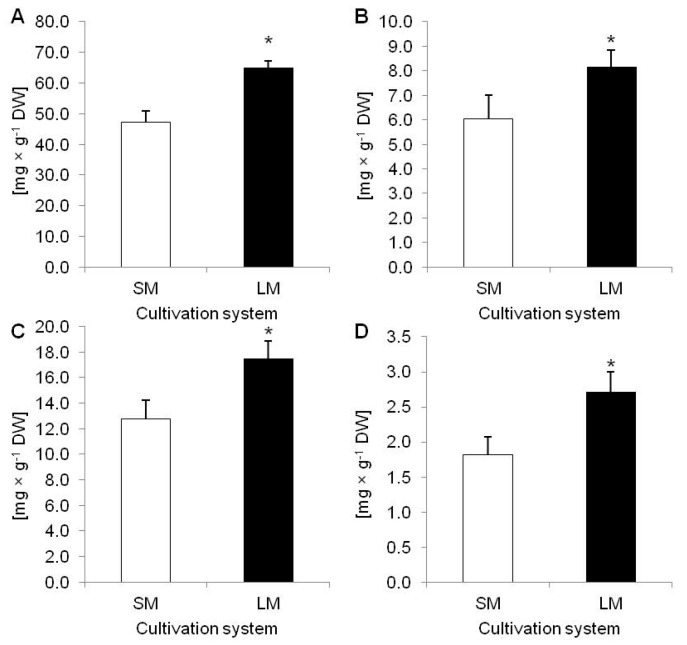
Accumulation of phenolic compounds in *Dionaea muscipula* plants cultivated on solid medium (SM) and in liquid medium with rotary shaking (LM); (**A**) total phenolic content; (**B**) phenylpropanoids; (**C**) flavonoids; and (**D**) anthocyanins depending on cultivation system; * significant difference between means at *p* < 0.05; bar—standard deviation.

To evaluate effect of LM on *D. muscipula* growth and development the growth index (GI) were calculated. Plants cultivated in LM had significantly higher GI (69.55%) than plants from SM (54.98%) (Figure 1A,B). In turn, dry weigh (DW) accumulation did not vary between tested techniques (Figure 1C). Moreover, LM with rotary shaking affected accumulation of phenolic compounds in plant tissue. In comparison to plants from SM, plants cultivated in LM accumulated significantly more: TPC, phenylpropanoids (PHE), flavonoids (FLA) and anthocyanins (ANT) (Figure 2A–D).

### 2.2. Results of D. muscipula Elicitation with C. sakazakii Lysate (Experiment 2)

**Figure 3:**
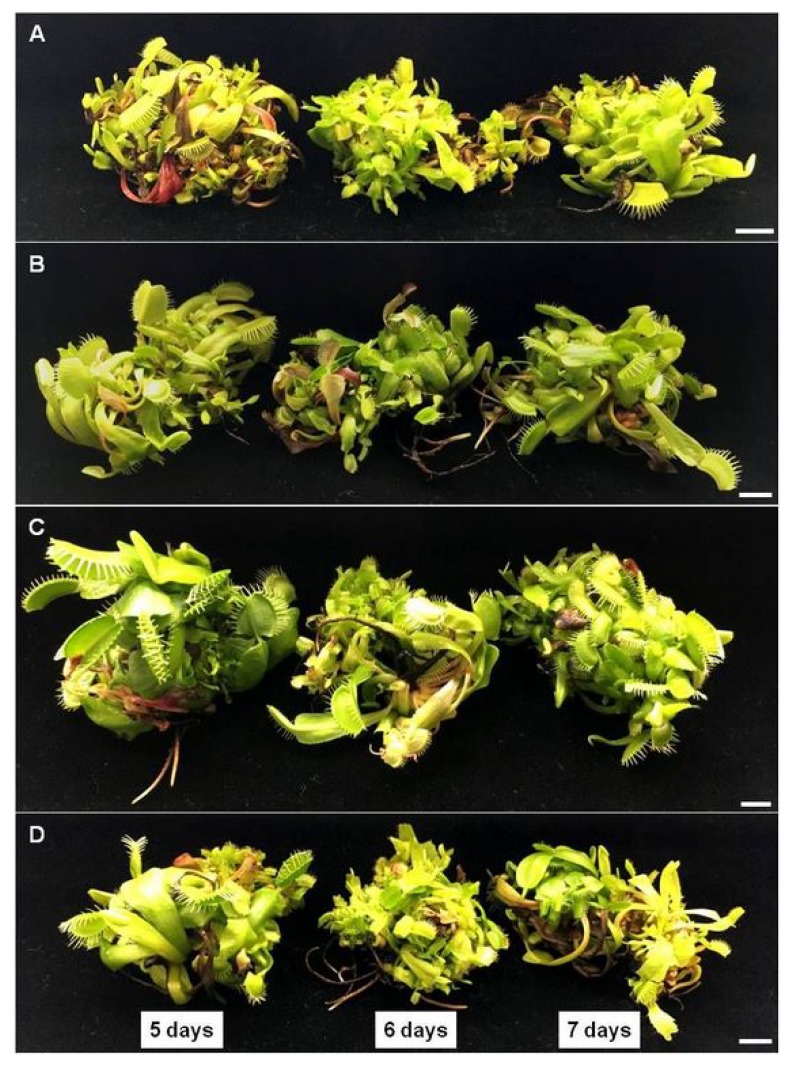
Morphology of *Dionaea muscipula* plants after 5, 6 and 7 days of elicitation with *Cronobacter sakazakii* lysate in different concentrations: (**A**) control (0%); (**B**) 1.5%; (**C**) 2.5%; (**D**) 5%; bar—1 cm.

**Figure 4:**
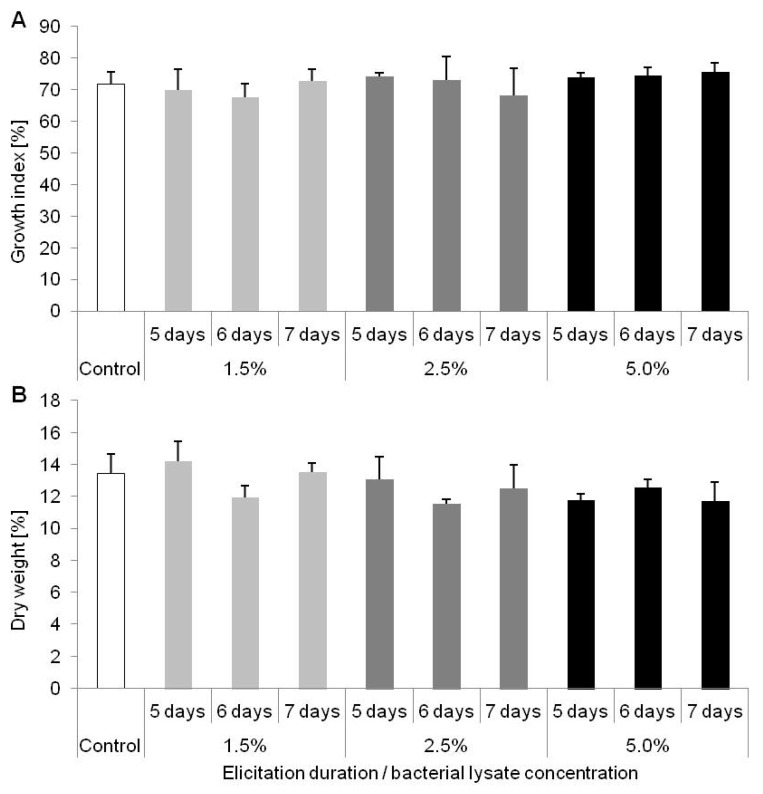
(**A**) Growth index (%) and (**B**) dry weight content (%) of *Dionaea muscipula* plants after 5, 6 and 7 days of elicitation with *Cronobacter sakazakii* lysate in different concentrations; no letters—no significant difference between means at *p* < 0.05; bar—standard deviation.

In this experiment we compared biometric parameters and accumulation of various phenolic derivatives in plants affected by biotic elicitation in comparison to untreated plants. In the Figure 3 A–D morphology of plants under various doses of elicitor (1.5; 2.5 or 5.0% of *C. sakazakii* lysate) and time of exposure (5, 6 or 7 days) is presented in comparison to untreated plants. The applied elicitor did not affect GI and DW content in *D. muscipula* (Figure 4A,B).

**Figure 5:**
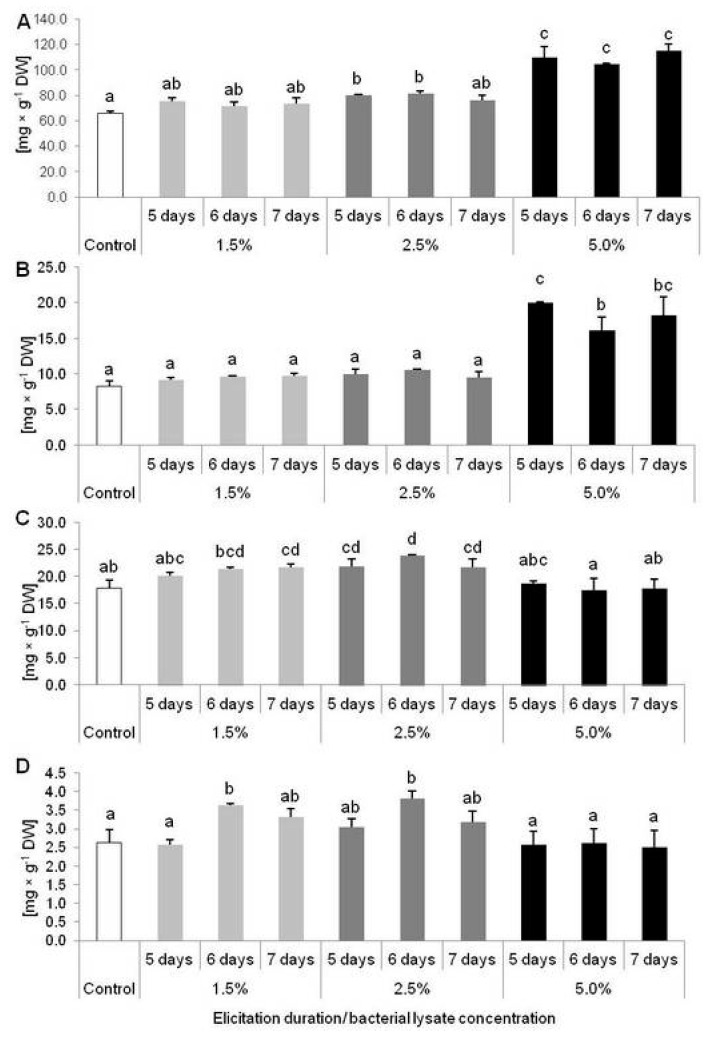
Accumulation of phenolic compounds in *Dionaea muscipula* plants after 5, 6 and 7 days of elicitation with *Cronobacter sakazakii* lysate in different concentrations: (**A**) total phenolic content; (**B**) phenylpropanoids; (**C**) flavonoids; (**D**) anthocyanins; different letters—significant difference between means at *p* < 0.05, bar—standard deviation.

In turn, TPC increased significantly in plants treated with 2.5% of elicitor after 5 and 6 days of exposure, as well as in those treated with 5% after 5, 6 and 7 days (Figure 5A). The highest level of TPC was noted in plants treated with 5% of bacterial lysate for 7 days, where concentration of phenolic compounds was 1.74-fold higher than in the control plants. Also, accumulation of PHE was affected by 5% of *C. sakazakii* lysate. After 5 days of treating, *D. muscipula* plants synthesized 2.43-fold more PHE than in control conditions (Figure 5B). On the other hand, FLA content grew significantly in plant tissues after 7 days of treatment with 1.5% and 2.5% of elicitor, regardless of the length of treatment (Figure 5C). Only lower doses of elicitor (1.5 and 2.5%) stimulated plants to higher ANT accumulation but only after 6 days of elicitation (Figure 5D).

**Figure 6:**
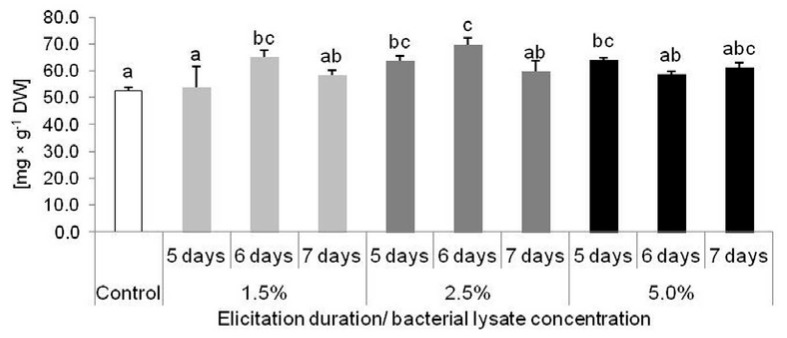
Accumulation of plumbagin in *Dionaea muscipula* plants after 5, 6 and 7 days of elicitation with *Cronobacter sakazakii* lysate in different concentrations; different letters—significant difference between means at *p* < 0.05; bar—standard deviation.

Applied elicitation also changed the accumulation of some phenolic derivatives in examined plants. Increased PLU content was noted after 5 days of treatment with 2.5% and 5% elicitor and after 6 days with 1.5% and 2.5% of the elicitor (Figure 6). The most effective results were noticed in plants treated with 2.5% elicitor for 6 days (69.82 mg × g^−1^ DW) (Figure 6).

**Figure 7:**
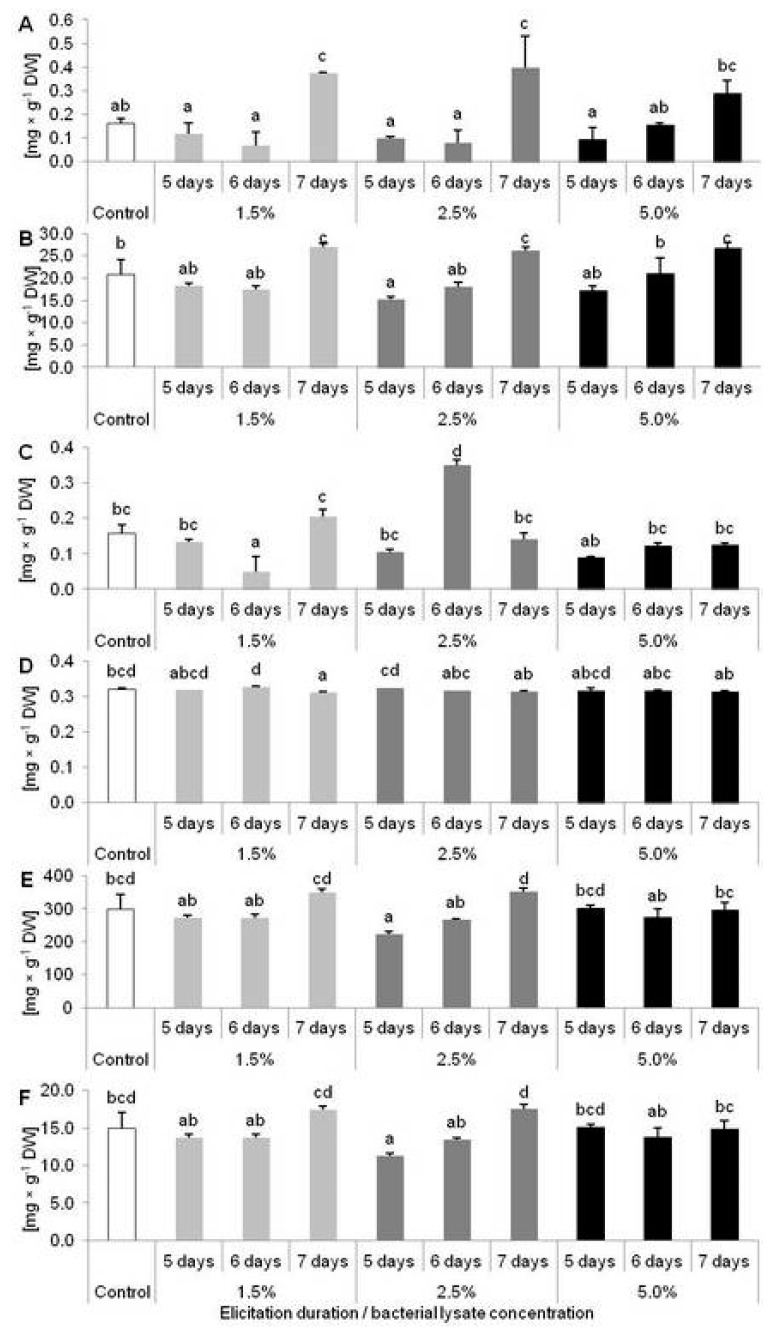
Accumulation of phenolic derivatives in *Dionaea muscipula* plants after five, six and seven days of elicitation with *Cronobacter sakazakii* lysate in different concentrations: (**A**) caffeic acid; (**B**) myricetin; (**C**) ellagic acid; (**D**) hyperoside; (**E**) salicylic acid; and (**F**) quercetin; different letters—significant difference between means at *p* < 0.05, bar—standard deviation.

Synthesis of caffeic acid (CA) significantly increased after 7 days of elicitation with 1.5 and 2.5% of the elicitor (2.31 and 2.43-fold higher synthesis, respectively) (Figure 7A). After 7 days of cultivation, accumulation of myricetin (MYR) also increased, not only in plants treated with 1.5 and 2.5% elicitor but also in those treated with 5% (Figure 7B). In turn, a higher level of ellagic acid (EA) was only recorded in plants treated with 2.5% elicitor for 6 days (Figure 7C). Interestingly, treatment with *C. sakazakii* lysate also caused a reduction of some phenolics accumulation (Figure 7B–F). MYR, salicylic acid (SA) and quercetin (QUE) content decreased in plants after 5 days of elicitation with 2.5% bacterial lysate (Figure 7 B,E,F). EA and hyperoside (HYP) accumulation were reduced after treatment with 1.5% of elicitor for 6 and 7 days, respectively (Figure 7C,D).

### 2.3. Biological Activity of Extracts from D. muscipula Plants Elicited with C. sakazakii Lysate

#### 2.3.1. Reactive Oxygen Species Scavenging Activity

**Figure 8:**
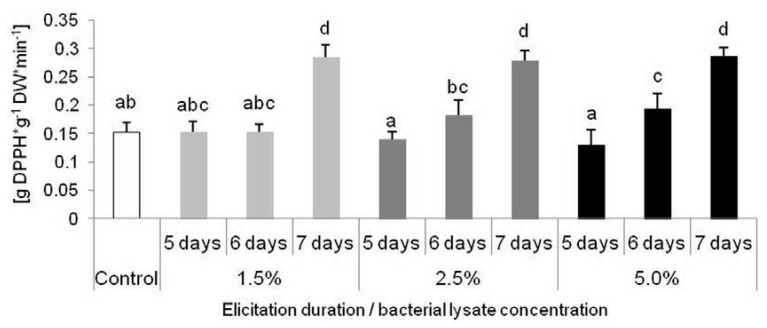
ROS scavenging activity (expressed as g DPPH reduced per g DW per minute) of *Dionaea muscipula* plants after 5, 6 and 7 days of elicitation with *Cronobacter sakazaki* lysate in different concentrations; different letters—significant difference between means at *p* < 0.05, bar—standard deviation.

The scavenging potential of extracts derived from *D. muscipula* tissue have been affected by some elicitor doses and exposure time. Significantly higher antioxidant activity have been noticed in plants treated 7 days by each concentration of elicitor, and also in plants treated 6 days with 5% of *C. sakazakii* lysate (Figure 8). 

#### 2.3.2. Antibacterial Activity

In the presented study, the MBC of plant tissue cultivated under bacterial elicitation have been investigated. Regardless of the elicitor concentration and exposure time, elicited plants extracts had 20% stronger activity against Gram-negative bacteria *E. coli* comparing to control plants (Table 1). 

Interestingly, in case of Gram-positive bacteria, *S. aureus,* the bactericidal properties of examined plants have been affected, depending on the elicitor concentration and time of elicitation. Tissue cultures treated with 1.5% of *C. sakazakii* lysate had 17% higher activity after 6 days of elicitation and about 34% increased bactericidal properties after 7 days of treatment. For treatment with 2.5 and 5% of elicitor the same effect has been observed (Table 1). After 5 and 6 days plant tissue had 17% higher activity against *S. aureus*, while after 7 days of treatment antibacterial strength increases 34% in compare to control plants.

## 3. Discussion

### 3.1. The Effect of Shaking on Plant Growth and Secondary Metabolite Levels

In the presented study, we focused on the possibility to produce a large amount of *D. muscipula* plant biomass with a high concentration of medical-active phenolic compounds. According to some authors, shaking technology has a lot of benefits for various types of cultures [29]. LM, with a rotary shaking system, is usually used for bacteria, cell suspensions or roots culture cultivation [29]. Due to fast oxygen and nutrient transfer from medium to living cells [29], lack of impurities from agar and the dilution of some exudates released from roots, like phenolic compounds [30], it is possible to obtain a faster growth and multiplication rate. This is the reason why, in the first experiment, we tested whether LM with rotary shaking will be a suitable system for Venus flytrap whole plants culture propagation. The results showed that such a cultivation system increased GI of the examined plants (by approx. 25%) in comparison to plants cultured on SM. Interestingly, our results are in agreement with observations by Liu et al. [31] that *Artemisia judaica* plants propagated in an LM flask culture with rotary shaking accumulated more FW and had higher proliferation rate than plants on SM. Also, Weathers et al. [32] revealed that *Artemisia annua* plants cultivated in LM with rotary shaking accumulated 25–50% more biomass than plants in bioreactors. Moreover, we conclude that LM conditions could be a good way for carnivorous plants to propagate because of their biology. In natural conditions, these plants occur in wet and flooded areas, which may result in a good acclimation mechanism to the physical properties of such a medium.

Once the culture conditions are optimized for higher biomass production of medical plants, the desired goal is to increase the amount of secondary metabolites in tissue. Unfortunately, these two objectives do not always occur in the tandem [32]. According to the results of Lattanzio et al. [33], under stress conditions, higher synthesis of phenolic compounds is strongly negatively correlated with the growth rate of plant tissue. This phenomenon results from the fact that a stress response is very costly for a plant and that acclimation to stress conditions requires a plant to use the basic metabolism products for a defense response consisting of secondary metabolites production. Carbon skeletons produced in primary metabolites pathways are distributed to secondary metabolites production pathways. Lattanzio et al. [34] showed that increased amounts of phenolic compounds in suspension culture of *Cynara cardunculus* growing under nutritional stress, was connected with a decrease of suspension culture biomass. However, the cultivation of *D. muscipula* plants in LM with rotary shaking, the increase of GI was simultaneous with significantly higher accumulation of TPC, PHE, FLA and ANT. It may result from the fact that the composition of the medium, used in our experiment, does not cause nutrient deficit and plants do not have to manage the limited resources of nutrients [17]. On the other hand, increased production of phenolic compounds in Venus flytrap plants, in LM with rotary shaking, may result from the fact that such a system of cultivation induces hydromechanical stress [29]. The carnivorous plant leaf-traps, being very sensitive to mechanical stimulation, can sense mechanical stimulus as the potential for catching the prey, while it is known that secondary metabolites from the phenolic compounds group play as protectants in the process of prey digestion [15]. Moreover, hydromechanical stress in an LM system is connected with the intensity of culture’ shaking. Perez-Hernandez et al. [35] examined suspension cell culture of medical plant *Sphaeralcea angustifolia* and revealed that cells grown with 200 rpm had the highest cell biomass and increased concentration of sphaeralcic acid. Growing cells with 100 rpm induces oxygen deficit stress, while 400 rpm negatively affects viability of cells, which was the consequence of hydrodynamic stress. Nevertheless, liquid cultures are one of the most important branches of tissue cultures in biotechnology of medical plants, and the selection of specific conditions is the crucial for both studying of plant response to stress factors and obtaining large quantities of plant material for medical purposes [36]. In the presented research, we proved that LM with rotary shaking is an effective system for *D. muscipula* propagation and synthesis of valuable, biologically active, phenolic compounds. This is the first report where shaking technology was used for propagation of a carnivorous plant from family Droseraceae. Based on the results from experiment 1, the culture conditions for experiment 2 were developed.

### 3.2. Impact of Biotic Elicitation on Plant Growth and Secondary Metabolite Levels

Many plant-derived chemicals, with importance in the pharmacological industry, can be overproduced in response to an external stimulus called an elicitor. As the elicitors do not act equally in every plant species, elicitation studies in various plants, with biological activity, need to be conducted [32]. On the other hand, this technique has some limitations. It has been shown that, despite the elicitor’s contribution to increasing synthesis of secondary metabolites in plant tissue, the vitality of the in vitro culture can decline, resulting in decreased growth rate or conduct to plant death [32]. Gadzovska et al. [37] revealed that elicitation of phenolic compounds, using jasmonic acid in *Hypericum perforatum* suspension culture caused increase of TPC and FLA production with a simultaneous decrease of cells’ viability. Jesionek et al. [20] showed that the elicitation of essential oil in a *Rhododendron tomentosum* bioreactor-grown microshoots culture, with aphid ethanol extract and bacteria lysates from *Candida albicans*, *C. sakazakii*, *Pectobacterium carotovorum* and *Dickeya dadantii* decreased the GI of plants, compared to untreated control shoots. In turn, *Ruta graveolens* shoots elicited with lysate of *Bacillus* sp. cells have been characterized by increased growth and accumulation of coumarin [38]. In the presented study, a tissue culture of *D. muscipula* was elicited in LM with rotary shaking using lysate from *C. sakazakii* cells, with various concentrations and exposure time to elicitors. Neither DW content nor GI was affected by elicitor treatments in comparison to control plants. It may be connected with a short time of elicitation (5, 6 or 7 days) and/or effective acclimation mechanisms of Venus flytrap plants for stress connected with bacterial elicitation. These results are in agreement with Krolicka et al. [1] findings, where FW of *D. muscipula* did not change under biotic elicitation with *Agrobacterium rhizogenes* lysate. On the other hand, in our previous studies, GI did not change in plants growing in higher light intensity or under white LED light in comparison to the fluorescence radiation [13], while blue-red LED light increased GI of *D. muscipula* and *Drosera peltata* cultivated in vitro [14]. 

Only few articles indicate the activity of lysate from *C. sakazakii* on the plant’s secondary metabolism. Previously, Staniszewska et al. [39] and Krolicka et al. [25] reported that the elicitation of *Ammi majus* with *C. sakazakii* led to changes in metabolism of cumarins and can decrease the growth rate of tissue culture. Jesionek et al. [20] did not find any changes of essential oil content in *Rhododendron* culture elicited with lysate from *C. sakazakii*. In our study, for the first time, we presented application of this elicitor for increased production of phenolic compounds in the *D. muscipula* plant. Krolicka et al. [1] showed that, lysate from *A. rhizogenes,* increased content of PLU in a Venus flytrap tissue culture, with simultaneous higher antibacterial activity of extract derived from elicited plants. Moreover, Krolicka et al. [1] reported that such an elicitation strategy did not affect the synthesis of FLA: MYR and QUE. We noticed that accumulation of total FLA, PLU and MYR was affected significantly by some of elicitor’s concentrations and exposure time, while the highest yield of PLU was obtained by a treatment with 2.5% of bacteria lysate for six days. Moreover, the highest TPC and PHE accumulation was obtained with 5% of *C. sakazakii* lysate, independently of the exposure time. In contrast, CA and MYR synthesis was affected by exposure time to the elicitor. The highest amount of these metabolites was obtained after seven days of elicitation, regardless the dose of bacterial lysate. Furthermore, elicitation with *C. sakazakii* led to decrease in content of some phenolic derivatives. After five days with 2.5% of bacteria lysate synthesis of SA, QUE and MYR decreased, after 6 days of treatment with 1.5% of elicitor level of EA decreased, while content of HYP was significantly lower after five days of treatment with 2.5% of elicitor. Such negative changes in secondary metabolites content can be also the consequence of the stress-related response of plants [20]. 

Based on these results, we can conclude that the chosen elicitor (containing the endotoxin O-antigen involved in bacterial pathogenesis) stimulates some of the phenolic derivatives in Venus flytraps; the response of this plant is also not specific and we cannot outline clear relationships between elicitation effect and content of all phenolic derivatives. It is worth noting that the O-antigen polysaccharide of the bacterial cell surface are mostly involved in a host specific immunological response [28] and clearly affects the content of secondary metabolites in Venus flytrap tissue. Moreover, flagellum protein synthesized by *C. sakazakii* is an important virulence factor for bacteria pathogenic to animals and plants [40], so it is possible that this protein acted as an elicitor in our study. Furthermore, we conclude that bacterial elicitation is a more suitable strategy to improve secondary metabolites production in *D. muscipula*, than light elicitation [13,14].

Other authors also showed some biotic elicitation strategies of phenolic compounds in different medical plant species. One of the most common agents used in elicitation of phenolic derivatives in plants is chitosan. Chitosan was reported to increase phytoalexin production in *R. graveolens* [41], stimulate lignans accumulation in *Schisandra chinensis* [19] and affect higher accumulation of TPC in *Orthosiphon stamineus* [42]. Moreover, the application of chitosan, yeasts extract or precursor feeding in elicitation of PLU in *Plumbago* roots cultures is popular [11,12,43,44]. Comparing these works to our results, elicited *D. muscipula* plants accumulate more PLU and other phenolic derivatives. This probably results from the specific acclimation strategies of carnivorous plants related to their ecophysiology and gives a basis for further studies on the metabolism of phenolic compounds in carnivorous plants. 

### 3.3. Impact of Biotic Elicitation on Biological Properties of D. muscipula Plants

In our study we evaluated how the biotic elicitation with *C. sakazakii* lysate affects biological activity of extracts derived from *D. muscipula* tissue culture. For the first time antioxidant properties of elicited Venus flytrap tissue culture were evaluated using method based on scavenging of DPPH free radical. It was reported previously by Krolicka et al. [5], that carnivorous plant tissue from family Droseraceae is very potent antioxidant. In our research, the highest activity against DPPH free-radicals were noticed in plants with increased accumulation of CA and MYR, which is in agreement with research by Banasiuk et al. [45]. The reduction potential of plant-derived extract is strictly correlated with the quantity of phenolic compounds, especially FLA. Banasiuk et al. [45] have reported, that water extracts from carnivorous plants with the highest concentration of flavonoids gives the best results in production of silver nanoparticles, while such feature is strictly related to their anti-oxidative potential. Similar findings have been shown by Ansari et al. [46], where transformed hairy root culture of *Ligularia fischeri* were characterized by increased synthesis of phenolic compounds and higher radical scavenging activity, than control plants. Moreover, *Cannabis sativa* cell suspension culture treated with jasmonates and some precursors of phenylpropanoid pathway accumulated more phenolic derivatives and had increased radical scavenging activity against DPPH [47]. 

Due to the rapid increase in human-pathogenic bacteria to antibiotic treatments, new sources of chemicals with strong antibacterial properties are needed [6]. Presented research was focused on potential use of extracts from elicited medical plant *D. muscipula* against antibiotic-resistant bacteria. In comparison to *D. muscipula* control culture growing without elicitation treatment, extracts from elicited plants had increased bactericidal activity against Gram-negative bacteria *E. coli*. Interestingly, regardless of exposure time and elicitor concentration antibacterial properties of examined plants increased 20%. It may be a consequence of lower sensitivity of Gram-negative bacteria to plant-derived metabolites, than Gram-positive bacteria [48], which is confirmed by the presented results, where MBC for Gram-positive bacteria *S. aureus* is dependent on duration of the elicitation. Krolicka et al. [1] showed, that elicitation with lysate of *A. rhizogenes* can stimulate antibacterial properties of *D. muscipula* tissue culture against Gram-negative bacteria *Klebsiella pneumonia*, while MBC for Gram-positive *S. aureus* was not changed. On the contrary, our elicitation strategy with lysate of *C. sakazakii* increased bactericidal properties of Venus flytrap tissue against this pathogen. Moreover, for the first time we can report that MBC of carnivorous plant tissue for *S. aureus* is dependent on elicitation treatment time. Furthermore, antibacterial activity of elicited plants does not correlate with the concentration of PLU, what can indicate, that not only accumulation of 1,4-naphtoquinones [5], but also quantity of others chemicals in carnivorous plants metabolic profile has crucial importance in potential healing properties of these plants.

## 4. Materials and Methods

### 4.1. Plant Material and Experiments Design

#### 4.1.1. Plant Material 

This study was conducted on a previously established in vitro culture of *D. muscipula* plants [1]. Plants were cultivated on ½ strength MS medium [49] with no growth regulators, 3% sucrose and pH = 5.5 (adjusted prior autoclaving) and solidified with 0.8% agar. Plants were cultivated at temperature 23 ± 1 °C; in fluorescence light of 80 μmol × m^−2^ × s^−1^ photosynthetic photon flux density (PPFD); (photoperiod 16 h/8 h light/dark cycle) and subcultured at 30-daya intervals.

#### 4.1.2. Experiment 1: Cultivation of Plants in Liquid Media with Rotary Shaking 

About 1.5 g of plants cultured as described above were subcultured to flasks with solid medium (SM) or liquid medium (LM). Chemical composition of media was the same as above. Tissue cultures in LM were put on a rotary shaker (130 rpm × min^−1^). After 30 days of cultivation, plants were subjected to growth parameters determination and biochemical analysis. There were 5 biological repetitions (flasks) of each variant (SM or LM) prepared, and the experiment was repeated in triplicate.

#### 4.1.3. Experiment 2: Elicitation of Plants Growing in Liquid Media with Rotary Shaking 

The elicitor in this experiment was lysate from human-pathogenic bacteria *Cronobacter sakazakii* ZOBR A741, and selected based on our preliminary data and available literature [39]. The elicitor was prepared according to Jesionek et al. [20]. Briefly, microbes were cultivated 24 h in Luria broth (LB) medium at 37 °C. Suspension cultures (15 × 10^12^ colony-forming units (CFU)/mL, according to McFarland scale) were treated with toluene (100:1 *v*/*v*), toluene was evaporated, and the elicitor was autoclaved. This prepared lysate was used for plant elicitation.

About 1.5 g of plant material was subcultured to flasks with LM (composition as described above) and placed on a rotary shaker (130 rpm × min^−1^). Tissue cultures were cultivated in the same light and temperature conditions as above. After 21 days of cultivation, lysate of *C. sakazakii* was added to media up to final concentrations: 1.5; 2.5 and 5.0%. The exposure time for each treatment was 5, 6 or 7 days, according to the literature [20,23]. The control in the experiment was non-treated plants cultivated in the same conditions. Plant materials were examined for biometric and biochemical parameters. For experiment 5, biological repetitions (flasks) of the control and each treatment with the elicitor were prepared. The experiment was repeated in triplicate.

### 4.2. Growth Parameters Estimation

#### Growth Index (GI) and Dry Weight (DW) Content

Plants from experiment 1 and 2 were weighed immediately after harvesting. Growth index (GI) was calculated according to formula: GI [%] = (FW_2_ − FW_1_)/FW_2_ × 100, where FW_1_ is fresh weight of plants at the beginning of experiment and FW_2_ is a final fresh weight. Next, plant material was freeze-dried for 72 h and weighed to determine content of dry weight (DW) using formula: DW [%] = DW_2_ × 100/FW_2_, where DW_2_ is dry weight after freeze-drying. Freeze-dried plant tissue was homogenised and stored at −20 ^o^C.

### 4.3. Biochemical Analysis 

#### 4.3.1. Spectrophotometric Estimation of Total Phenolic Content (TPC)

TPC was assessed using Folin-Ciocalteu’s reagent [50], with modifications according to Makowski et al. [14]. In short, 10 mg of freeze-dried plant material was extracted in 1 mL of 80% methanol at 4 °C. Samples were centrifuged for 15 min (25,155 g, 4 °C). Of the diluted extract, 1 mL was mixed with 0.2 mL of Folin’s reagent (Sigma-Aldrich Chemie, GmBH, Stein-heim, Germany), 1.6 mL of 5% Na_2_CO_3_ and incubated for 20 min at 40 °C. The absorbance of samples was measured at 740 nm, using a Double Beam spectrophotometer U-2900 (Hitachi High-Technologies Corporation). Chlorogenic acid (Sigma-Aldrich Chemie, GmBH, Steinheim, Germany) was used as a reference standard. Results were expressed as milligram of chlorogenic acid equivalents per 1 g of DW. Analyses were done in 5 replicates.

#### 4.3.2. Spectrophotometric Estimation of Phenylpropanoids (PHE), Flavonoids (FLA) and Anthocyanins (ANT) Content

PHE, FLA and ANT accumulation were estimated using the method of Fukumoto and Mazza [51], with modifications [13]. Plant tissue was extracted like in the method for TPC estimation. Supernatant was mixed with 0.25 mL 0.1% HCl in 96% EtOH and 4.55 mL 2% HCl in H_2_O. Samples were incubated at room temperature (darkness) for 20 min. Absorbance was measured at wavelengths of 320, 360 and 520 nm. Contents of PHE, FLA and ANT were calculated using calibration curves made for caffeic acid, quercetin and cyanidin (Sigma-Aldrich Chemie, GmBH, Steinheim, Germany), respectively. The results were expressed as milligram of standard equivalents per 1 g of DW. Analyses were done in 5 replicates.

#### 4.3.3. High Pressure Liquid Chromatography (HPLC) Analysis of Phenolic Compounds

For analysis of PLU content, freeze-dried plant tissue was extracted in 0.5 mL of redistilled H_2_O and 0.5 mL of tetrahydrofuran (THF) according to Tokarz et al. [52]. To extract other phenolic derivatives, like caffeic acid (CA), hyperoside (HYP), ellagic acid (EA), salicylic acid (SA), myricetin (MYR) and quercetin (QUE), 20 mg of dry tissue was homogenised in 2 mL of 100% methanol (4 °C) and sonicated for 30 min. Samples were centrifuged for 15 min (25,155 g, 4 °C). Supernatant was collected for chromatographic analysis (HPLC).

The chromatographic separation was carried out using Dionex UltiMate 3000 HPLC system equipped with a quaternary pump, autosampler, column oven and UV detector. For the stationary phase, an Agilent Zorbax SB-Phenyl (4.6 × 150 mm, 3.5 µm) was used. The flow rate used was 1 mL × min^−1^. The sample injection volume was 10 µL. The mobile phase for the analysis consisted of 0.1% (*v/v*) trifluoroacetic acid in acetonitrile as eluent A and 0.1% (*v/v*) trifluoroacetic acid in water as eluent B. The separation gradient was 0 min (10% A)-> 5 min (10% A)-> 12 min (90% A)-> 20 min (90% A), followed by a 10-min column regeneration. Chromatographic separations were carried out at 25 °C. Typical compounds present in carnivorous plant tissues (plumbagin, hyperoside, ellagic acid, myricetin, quercetin, salicylic acid and caffeic acid) were used as standards to determine extract composition. A three-level standard curve was used for determining the concentration of the compounds 4-point. Monitoring was performed at 254 nm. All analyses were performed in triplicate.

### 4.4. Analysis of Biological Activity of Examined Plants

#### 4.4.1. Spectrophotometric Estimation of Antioxidative Properties of Plant Extract Using DPPH Method

Scavenging of 2,2-diphenyl-1-picryl-hydrazyl (DPPH) free radical was measured using the methods of Sharma and Bhat [53] and Elshafie et al. [54], with modifications. Plant tissue was extracted like in the method for TPC estimation. 0.05 mL of diluted methanolic extract was mixed with 2.95 mL of 50 mM DPPH solution and incubated in the dark. After 30 min absorbance of samples were measured at 517 nm. The reduction of stable DPPH by plant extract was expressed as a gram of DPPH reduced by gram of DW tissue per one minute. Analyses were done in five replicates.

#### 4.4.2. Antibacterial Activity

The antibacterial properties of the examined plants were evaluated using a minimal bactericidal concentration (MBC) method by Krolicka et al. [5]. MBC was determined against antibiotic-resistant bacteria: *Staphylococcus aureus* ATCC 25923 G (+) and *Escherichia coli* ATCC 25922 G (−), obtained from Intercollegiate Faculty of Biotechnology, University of Gdańsk and Medical University of Gdańsk, Poland. The bacteria were cultivated overnight on BHI medium at 37 °C, before the tests. Plant tissue (100 mg DW) was extracted in THF [13]. Extracts were evaporated and resuspended in methanol before application into wells of the 96-well plate. After application, extracts were evaporated to remove toxic for bacteria methanol. The residues were suspended in 100 μL liquid BHI medium for bacteria cultivation and aliquots of 10 μL of the bacterial suspension (10^5^ CFU × ml^−1^) in liquid medium was added into wells. Plates were incubated overnight. In order to establish the MBC value, 100 μL of the content of each well that were shown no visible growth of bacteria were plated out on an BHI agar plate, for 24 h incubation at 37 °C. The MBC was defined as the lowest concentration of the extract that reduced the inoculum by 99.9% within 24 h.

### 4.5. Statistical Analyses

Results from experiment 1 were subjected to Student’s T-test with *p <* 0.05 level. In experiment 2, one-way analysis of variance (ANOVA) was used to determine significant differences between means (Tukey test at *p <* 0.05 level). STATISTICA 12.0 (StatSoft Inc., Tulsa, OK, USA) was used to carry out statistical analyses.

## 5. Conclusions

The presented research enabled us to study growth and accumulation of pharmacologically active phenolic compounds in a carnivorous *D. muscipula* plant tissue culture, using liquid medium with rotary shaking system and biotic elicitation. The results in experiment 1 proved that liquid media, with rotary shaking, are promising for bigger scale use in, not only the scientific, but also the industrial field. Due to the sensitivity of carnivorous plants to mechanical stimulation, hydromechanical stress in shaking cultures makes it possibility to increase the content of defense compounds. Furthermore, we conclude that lysate from *C. sakazakii* can be a useful elicitor for some of phenolic compounds in *D. muscipula* tissue cultures and it increases biological activity of *D. muscipula* plants against ROS and highly antibiotic-resistant, human-pathogenic bacteria. We can conclude, that examined plant is strongly antioxidant potent and increasing of radical scavenging activity of Venus flytrap tissue is the most dependent on the concentration of compounds, like PHE and FLA. Also, elicitation with lysate of *C. sakazakii* turned out to be a useful tool for enhancing of antibacterial activity for both: Gram-positive and Gram-negative pathogens. These report gives bases for further investigations on carnivorous plants from family Droseraceae in medical plant biochemistry and pharmacology. 

## Figures and Tables

**Table 1:** Minimal bactericidal concentration (MBC) of *S. aureus* and *E. coli* after treatment with extracts of *Dionaea muscipula* plants after five, six and seven days of elicitation with *Cronobacter sakazaki* lysate in different concentrations.

Concentration of *C. sakazaki* Lysate (%)	Days of Elicitation	*Staphylococcus aureus* ATCC 25923	*Escherichia coli* ATCC 25922
		MBC (µg DW × mL^−1^)	
0.0 (Control)		501	2087.5
1.5	5	501	1670
	6	417.5	1670
	7	334	1670
2.5	5	417.5	1670
	6	417.5	1670
	7	334	1670
5.0	5	417.5	1670
	6	417.5	1670
	7	334	1670

## Abbreviations

ANT	Anthocyanins
CA	Caffeic acid
CFU	Colony-forming unit
DPPH	2,2-diphenyl-1-picryl-hydrazyl
DW	Dry weight
EA	Ellagic acid
FLA	Flavonoids
GI	Growth index
HYP	Hyperoside
LM	Liquid medium
MBC	Minimal Bactericidal Concentration
MYR	Myricetin
PHE	Phenylpropanoids
PLU	Plumbagin
PPFD	Photosynthetic photon flux density
QUE	Quercetin
SA	Salicylic acid
SM	Solid medium
THF	Tetrahydrofuran
TPC	Total phenolic content
